# Biochemical characterization of the placeholder nucleosome for DNA hypomethylation maintenance

**DOI:** 10.1016/j.bbrep.2019.100634

**Published:** 2019-04-10

**Authors:** Rina Hirano, Tomoya Kujirai, Lumi Negishi, Hitoshi Kurumizaka

**Affiliations:** a Laboratory of Chromatin Structure and Function, Institute for Quantitative Biosciences, The University of Tokyo, 1-1-1 Yayoi, Bunkyo-ku, Tokyo, 113-0032, Japan; b Department of Biological Sciences, Graduate School of Sciences, The University of Tokyo, 1-1-1 Yayoi, Bunkyo-ku, Tokyo, 113-0032, Japan; c Graduate School of Advanced Science and Engineering, Waseda University, 2-2 Wakamatsu-cho, Shinjuku-ku, Tokyo, 162-8480, Japan

**Keywords:** DNA methylation, H2A.Z, H3K4 methylation, Nucleosome, Histone, Chromatin

## Abstract

DNA methylation functions as a prominent epigenetic mark, and its patterns are transmitted to the genomes of offspring. The nucleosome containing the histone H2A.Z variant and histone H3K4 mono-methylation acts as a “placeholder” nucleosome for DNA hypomethylation maintenance in zebrafish embryonic cells. However, the mechanism by which DNA methylation is deterred by the placeholder nucleosome is poorly understood. In the present study, we reconstituted the placeholder nucleosome containing histones H2A.Z and H3 with the Lys4 mono-methylation. The thermal stability assay revealed that the placeholder nucleosome is less stable than the canonical nucleosome. Nuclease susceptibility assays suggested that the nucleosomal DNA ends of the placeholder nucleosome are more accessible than those of the canonical nucleosome. These characteristics of the placeholder nucleosome are quite similar to those of the H2A.Z nucleosome without H3K4 methylation. Importantly, the linker histone H1, which is reportedly involved in the recruitment of DNA methyltransferases, efficiently binds to all of the placeholder, H2A.Z, and canonical nucleosomes. Therefore, the characteristics of the H2A.Z nucleosome are conserved in the placeholder nucleosome without synergistic effects on the H3K4 mono-methylation.

## Highlights

• The placeholder nucleosome containing H2A.Z and H3K4me1 was reconstituted *in vitro*.
• The placeholder nucleosome has similar characteristics to the H2A.Z nucleosome.
• H3K4me1 may not affect the stability and structure of the placeholder nucleosome.

## 1 Introduction

Genomic DNA is highly organized as chromatin in eukaryotes. The nucleosome is the basic architecture of chromatin. In the nucleosome, the histone proteins, H2A, H2B, H3, and H4, form a complex referred to as a histone octamer, which continuously binds 140–150 base pairs of DNA on its surface [1,2]. The nucleosomes are connected with linker DNA segments, and form a beads-on-a-string structure in chromatin [3].

The chromatin architecture is basically inhibitory for nuclear events, such as transcription. Nucleosome formation interferes with the DNA binding of transcription regulatory proteins, such as activators and repressors [[4], [5], [6]]. The nucleosome is also an obstacle for transcription by RNA polymerases [6,7]. In the nucleosome template, RNA polymerase II produces an RNA product at a reduced rate, as compared to a naked DNA template, by peeling the nucleosomal DNA in a stepwise manner from the histone surface [8,9]. Therefore, the appropriate amounts and positions of nucleosome formation are the key elements for proper gene regulation.

The nucleosome characteristics are dictated by histone variants and histone post-translational modifications (PTMs), which confer versatility to the nucleosome structure and stability [10]. Various histone variants are encoded in genomes, and are usually incorporated into chromatin in a cell-cycle independent manner [11,12]. In contrast, canonical histones are produced during the S-phase of the cell cycle, and are incorporated in a DNA replication-coupled manner [11,12]. Numerous histone PTMs, such as lysine/arginine methylation, lysine alkylation, lysine ubiquitination, and serine/threonine phosphorylation, have also been identified in the solvent accessible histone tails and inaccessible histone-fold domains in the nucleosome [13,14]. These histone variants and PTMs in the nucleosome function as epigenetic marks, which confer the chromatin states to daughter cells and offspring [15,16].

In addition to histone variants and PTMs, DNA methylation is an essential epigenetic mark [17]. In zebrafish, the paternal DNA methylation patterns are reportedly inherited and maintained in transcriptionally quiescent cleavage embryos, whereas the maternal DNA methylation patterns are rearranged to the paternal patterns. Interestingly, the genomic DNA regions lacking DNA methylation are occupied by a placeholder nucleosome, containing a histone variant, H2A.Z, and H3K4 mono-methylation (H3K4me1) [18]. However, the mechanism by which the placeholder nucleosome suppresses the DNA methylation has remained unclear, because the physical and biochemical characteristics of the placeholder nucleosome have not been analyzed. In the present study, we prepared the placeholder nucleosome with human recombinant histones, and tested its biochemical properties by thermal stability, micrococcal nuclease sensitivity, DNaseI sensitivity, and linker histone H1 binding assays.

## 2 Materials and methods

### 2.1 Histones and nucleosome preparation

Human histones were prepared as described previously [19]. The human linker histone H1.2 was bacterially produced as a recombinant protein, and was purified as described previously [20]. To obtain histone H3 with the Lys4 methylation, the H3.2 K4C/C110A mutant was prepared. In the H3.2 K4C/C110A mutant, the Lys4 and Cys110 residues were replaced by Cys and Ala, respectively. The Cys residue inserted at position 4 of H3 was then alkylated [21], and H3.2 with a monomethyl-lysine analog, N-monomethyl-aminoethylcysteine, at position 4 (referred to as H3K4_C_me1 in this report) was prepared [22]. We analyzed the H3K4_C_me1 peptide by mass spectrometry, and confirmed that the modified peptide containing the H3K4_C_me1 residue, but little unmodified peptide, was detected. This suggested that the H3K4 mono-methylation was nearly complete. The nucleosomes were reconstituted with the 147 base-pair Widom 601 sequence [23,24] by the salt dialysis method, as described previously [25]. The resulting nucleosomes were purified by native polyacrylamide gel electrophoresis, using a Prep Cell model 491 apparatus (Bio-Rad).

### 2.2 Thermal stability assay of nucleosomes

The thermal stability assay was performed by the previously described method [26]. The fluorescence signal from SYPRO Orange, which hydrophobically binds to thermally denatured histones released from the nucleosome, was monitored. The nucleosome (equivalent to 0.225 μg DNA/μL) was incubated in 19 μL of 18 mM Tris–HCl (pH 7.5) buffer, containing 0.9 mM dithiothreitol (DTT), 100 mM NaCl, and SYPRO Orange (×5). The fluorescence signals were detected with a StepOnePlus Real-Time PCR unit (Applied Biosystems). A temperature gradient (26–95 °C, in steps of 1 °C/min) was employed. Fluorescence data were normalized to % values as (F(T)-F(26 °C))/(F(95 °C)-F(26 °C)). The terms F(T), F(26 °C), and F(95 °C) indicate each fluorescence value at a particular temperature, 26 °C, and 95 °C, respectively.

### 2.3 MNase digestion assay

The nucleosomes (206 nM) were incubated with micrococcal nuclease (MNase, 10 units/mL, Takara) at 37 °C for 0, 3, 6, 12, and 15 min, in 50 mM Tris–HCl (pH 8.0) buffer, containing 2.5 mM CaCl_2_, 25 mM NaCl, and 1.0 mM dithiothreitol. To terminate the reaction, 15 μL of deproteinization solution (200 mM Tris–HCl (pH 8.0), 80 mM EDTA, 0.5 mg/mL proteinase K (Roche), and 0.25% sodium dodecyl sulfate) was added to 10 μL of the reaction mixture. The resulting DNA fragments were analyzed by 8% native polyacrylamide gel electrophoresis with ethidium bromide staining.

### 2.4 DNaseI digestion assay

The nucleosomes (206 nM) were incubated with DNaseI (0.1 unit/mL, Takara) at 26 °C for 0, 2, 4, 6, 8, and 10 min, in 50 mM Tris–HCl (pH 8.0) buffer, containing 2.5 mM MgCl_2_, 25 mM NaCl, and 1.9 mM dithiothreitol. To terminate the reaction, 5 μL of deproteinization solution (20 mM Tris–HCl (pH 8.0), 20 mM EDTA, 0.25 mg/mL proteinase K (Roche), and 0.1% sodium dodecyl sulfate) was added to 10 μL of the reaction mixture. After deproteinization, 7 μL of Hi-Di formamide was added to 3 μL of the reaction mixtures, and the mixtures were heated at 95 °C to denature the DNA fragments. The resulting DNA fragments were analyzed by 8% polyacrylamide urea denaturing gel electrophoresis with SYBR Gold staining.

### 2.5 The linker histone H1 binding assay

The indicated amount of purified histone H1.2 was mixed with the placeholder, H2A.Z, or canonical nucleosome (0.1 μM), which was reconstituted with the 193 base-pair Widom 601 DNA in the presence of Nap1 (0.3 μM), in 10 μL of reaction buffer (35 mM Tris-HCl (pH 7.5), 70 mM NaCl, 0.01 mM PMSF, 0.05 mM EDTA, 6.5% glycerol, 1.2 mM dithiothreitol, and 1.1 mM 2-mercaptoethanol). The H1-nucleosome complexes were assembled in the mixture by an incubation at 25 °C for 30 min, and were separated by native 5% polyacrylamide gel electrophoresis in 1× TBE buffer (90 mM Tris base, 90 mM boric acid, and 2 mM EDTA). The gel was stained with ethidium bromide, and the bands were visualized with an LAS-4000 image analyzer (GE Healthcare).

## 3 Results and discussion

### 3.1 Reconstitution of the placeholder nucleosome containing H2A.Z and H3K4_C_me1

**Fig. 1:**
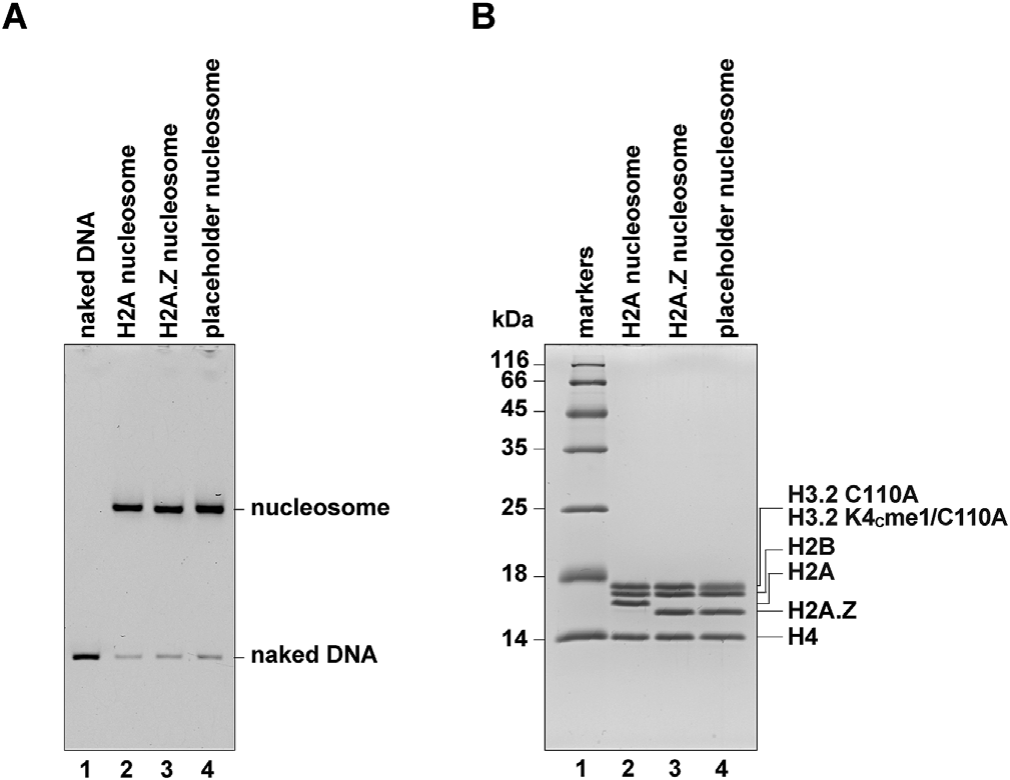
Preparation of the placeholder nucleosome. **A.** The purified nucleosomes were analyzed by native-PAGE with ethidium bromide staining. **B.** The purified nucleosomes were analyzed by SDS-PAGE with Coomassie Brilliant Blue staining.

To prepare the H3K4me1 peptide, we installed a monomethyl-lysine analog, N-monomethyl-aminoethylcysteine, at position 4 of H3.2. To do so, the H3.2K4 residue was replaced by Cys, and the Cys residue was alkylated to generate the monomethyl-lysine analog, N-monomethyl-aminoethylcysteine. The human histone H3.2 was employed as the canonical H3, because it contains only one Cys residue at position 110. To mask the H3.2 Cys110 residue from the alkylation reaction, it was replaced by Ala (H3.2 C110A). We then reconstituted three types of nucleosomes, the H2A nucleosome, the H2A.Z nucleosome, and the placeholder nucleosome, each containing the canonical H2A and H3.2 C110A, H2A.Z and H3.2 C110A, and H2A.Z and H3.2C110A with the N-monomethyl-aminoethylcysteine at position 4, respectively. In this study, H3.2 C110A and H3.2 C110A with the N-monomethyl-aminoethylcysteine at position 4 are referred to hereafter as H3 and H3K4_C_me1, respectively. The nucleosomes were reconstituted by the salt dialysis method, and were purified by native polyacrylamide gel electrophoresis (Fig. 1A). The purified nucleosomes contained all four histones in a stoichiometric manner (Fig. 1B).

### 3.2 Thermal stability of the placeholder nucleosome

**Fig. 2:**
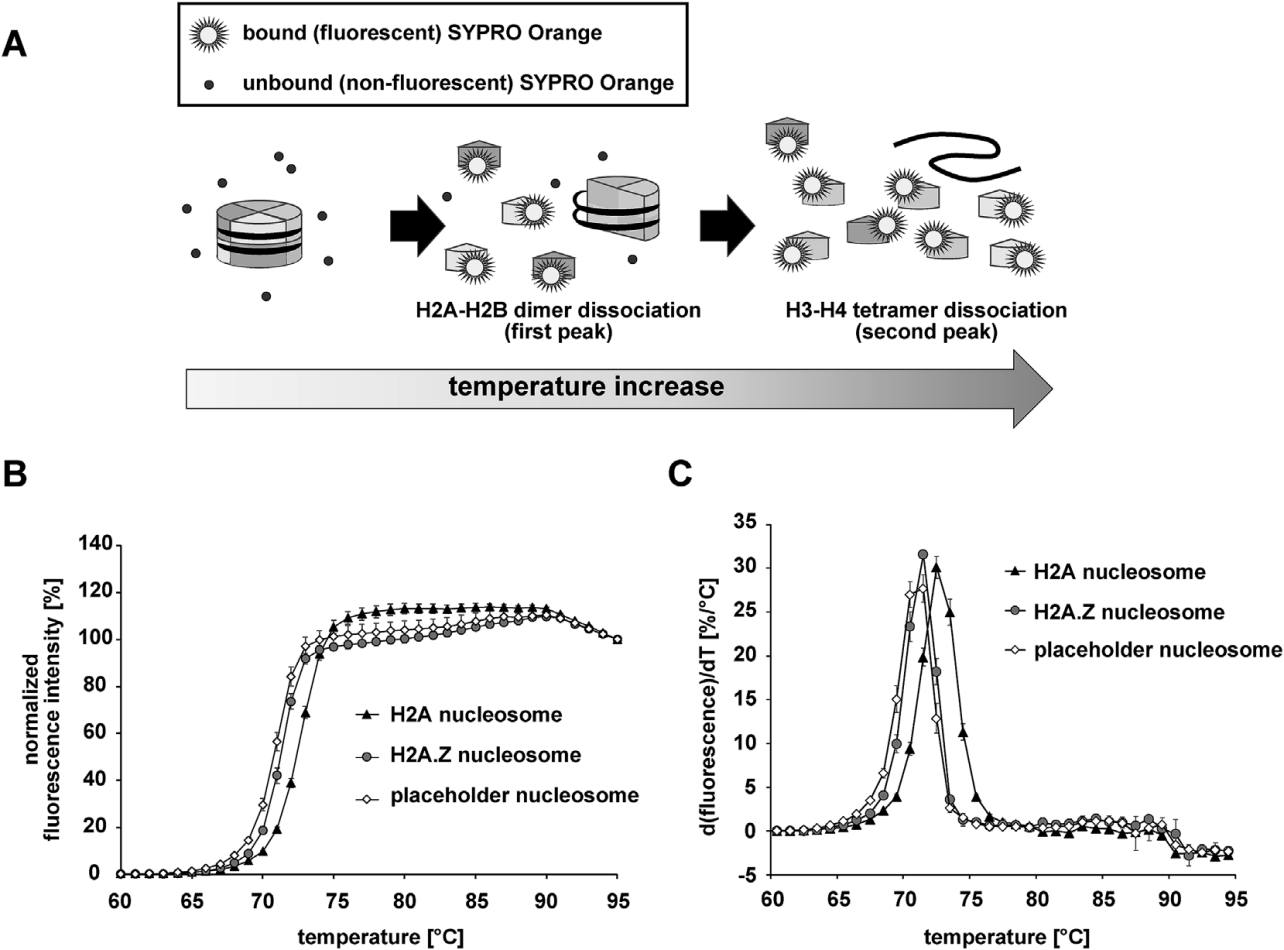
Thermal stability of the placeholder nucleosome. **A.** Schematic representation of the thermal stability assay. In this assay, thermal dissociations of the histones from the nucleosomes are detected using SYPRO Orange fluorescent dye, which emits substantial fluorescence when it binds to the exposed hydrophobic surfaces of the denatured histones. **B.** Normalized fluorescent intensity curves of thermal dissociations of the histones from the nucleosomes. The error bars indicate standard deviations. The experiments were performed four times. **C.** Plots of the derivatives of the curves in panel B.

To study the nucleosome stability, we performed a thermal stability assay. In this assay, the purified nucleosomes were denatured by heating, and the histones released from the nucleosome were detected with the fluorescent dye, SYPRO Orange [26]. Since SYPRO Orange binds to the hydrophobic surfaces of denatured proteins, it binds to the denatured free histones [26]. The fluorescence signals from the SYPRO Orange bound to the denatured histones were detected (Fig. 2A). We previously reported that the nucleosome reconstituted with the α-satellite DNA sequence showed a bi-phasic denaturation curve, in which the first and second phases correspond to the H2A-H2B dissociation and the H3-H4 dissociation from the nucleosome, respectively [26].

In the present study, we reconstituted the nucleosomes with the 147 base-pair Widom 601 sequence, which is a DNA sequence conferring high stability to the nucleosome [23]. As shown in Fig. 2B, the canonical nucleosome with the Widom 601 sequence exhibited a denaturation curve with the first phase around 70–75 °C, but the second phase was not clear as compared to the previously published curve with the α-satellite DNA sequence [26]. The placeholder and H2A.Z nucleosomes were less stable than the canonical nucleosome (Fig. 2B and C). There was little difference in the stability between the placeholder and H2A.Z nucleosomes, indicating that H3K4_C_me1 does not contribute to the nucleosome stability.

### 3.3 Accessibility of the nucleosomal DNA in the placeholder nucleosome

**Fig. 3:**
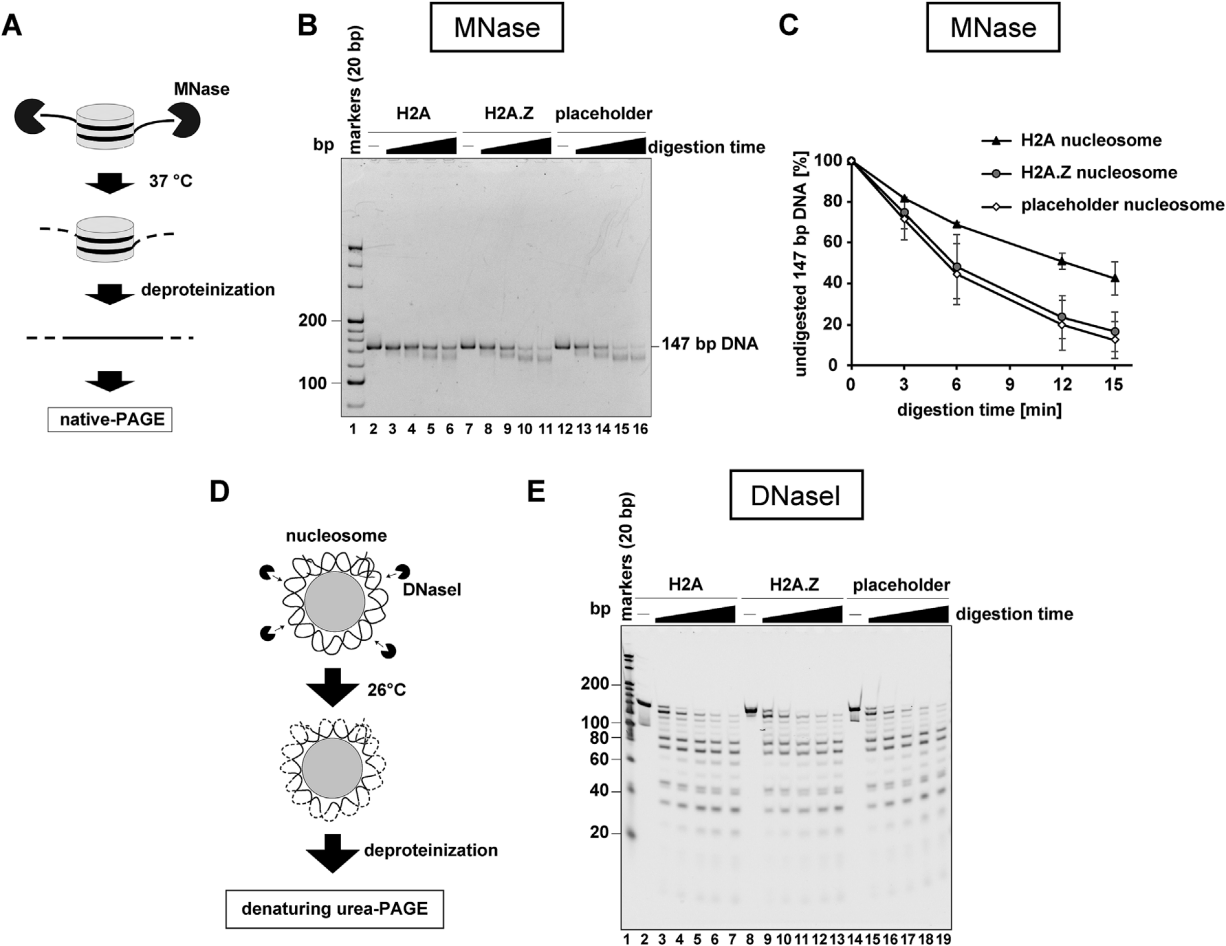
Accessibility of the nucleosomal DNA in the placeholder nucleosome. **A.** Schematic representation of the MNase susceptibility assay. In this assay, DNA ends detached from histones in the nucleosomes were preferentially digested by MNase. After the digestion reaction, the resulting DNA fragments were analyzed by native-PAGE. **B.** A representative gel image of the MNase susceptibility assay. The canonical (lanes 2–6), H2A.Z (lanes 7–11), and placeholder (12–16) nucleosomes containing the 147 base-pair Widom 601 DNA were treated with MNase for 0, 3, 6, 12, and 15 min at 37 °C. After the incubation, the reaction was stopped by adding stop solution containing proteinase K, SDS, and EDTA. The resulting DNA fragments were analyzed by native-PAGE with ethidium bromide staining. **C.** Graphic representation of the MNase susceptibility assay. The experiments were repeated three times, and the average values were plotted with the standard deviation values. **D.** Schematic representation of the DNaseI treatment assay. In this assay, the DNA strands exposed to the solvent are preferentially digested by DNaseI. After the digestion reaction, the resulting DNA fragments are analyzed by denaturing urea-PAGE. **E.** A representative gel image of the DNaseI treatment assay. The canonical (lanes 2–7), H2A.Z (lanes 8–13), and placeholder (lanes 14–19) nucleosomes containing the 147 base-pair Widom 601 DNA were treated with DNaseI for 0, 2, 4, 6, 8, and 10 min at 26 °C. The digestion reaction was stopped by adding stop solution containing proteinase K, SDS, and EDTA. The resulting DNA fragments were denatured and analyzed by urea-PAGE with SYBR Gold staining.

We then tested the flexibility and accessibility of the DNA in the placeholder nucleosome. To do so, we performed a micrococcal nuclease (MNase) treatment assay. MNase preferentially digests the DNA region freed from histones, while the DNA regions wrapped tightly around the histones in the nucleosome are not digested. Therefore, the DNA ends of the nucleosome are usually susceptible to MNase, and their flexibility can be evaluated by the MNase susceptibility (Fig. 3A). The DNA ends of the human H2A.Z nucleosome are more susceptible than those of the canonical nucleosome [27]. Interestingly, the DNA ends of the placeholder nucleosome were clearly more susceptible to MNase than those of the canonical nucleosome, suggesting their flexible nature (Fig. 3B and C). This characteristic may not be a consequence of the H3K4_C_me1, because the difference between the placeholder and H2A.Z nucleosomes was marginal (Fig. 3B and C).

We next tested the accessibility of the nucleosomal DNA region tightly wrapped around the histones. To do so, we performed the DNaseI treatment assay. DNaseI preferentially cut the DNA strand exposed to the solvent (Fig. 3D). Therefore, the histone-DNA contacts and the DNA strand paths in the nucleosome can be evaluated by the DNaseI cleavage pattern. As shown in Fig. 3E, there were no obvious differences among the placeholder, H2A.Z, and canonical nucleosomes in terms of the DNaseI cleavage patterns. Therefore, in the placeholder nucleosome, the DNA ends are more flexible than those in the canonical nucleosome, but this does not affect the major histone-DNA contacts within the placeholder nucleosome and the H2A.Z nucleosome.

### 3.4 Linker histone H1 binding to the placeholder nucleosome

**Fig. 4:**
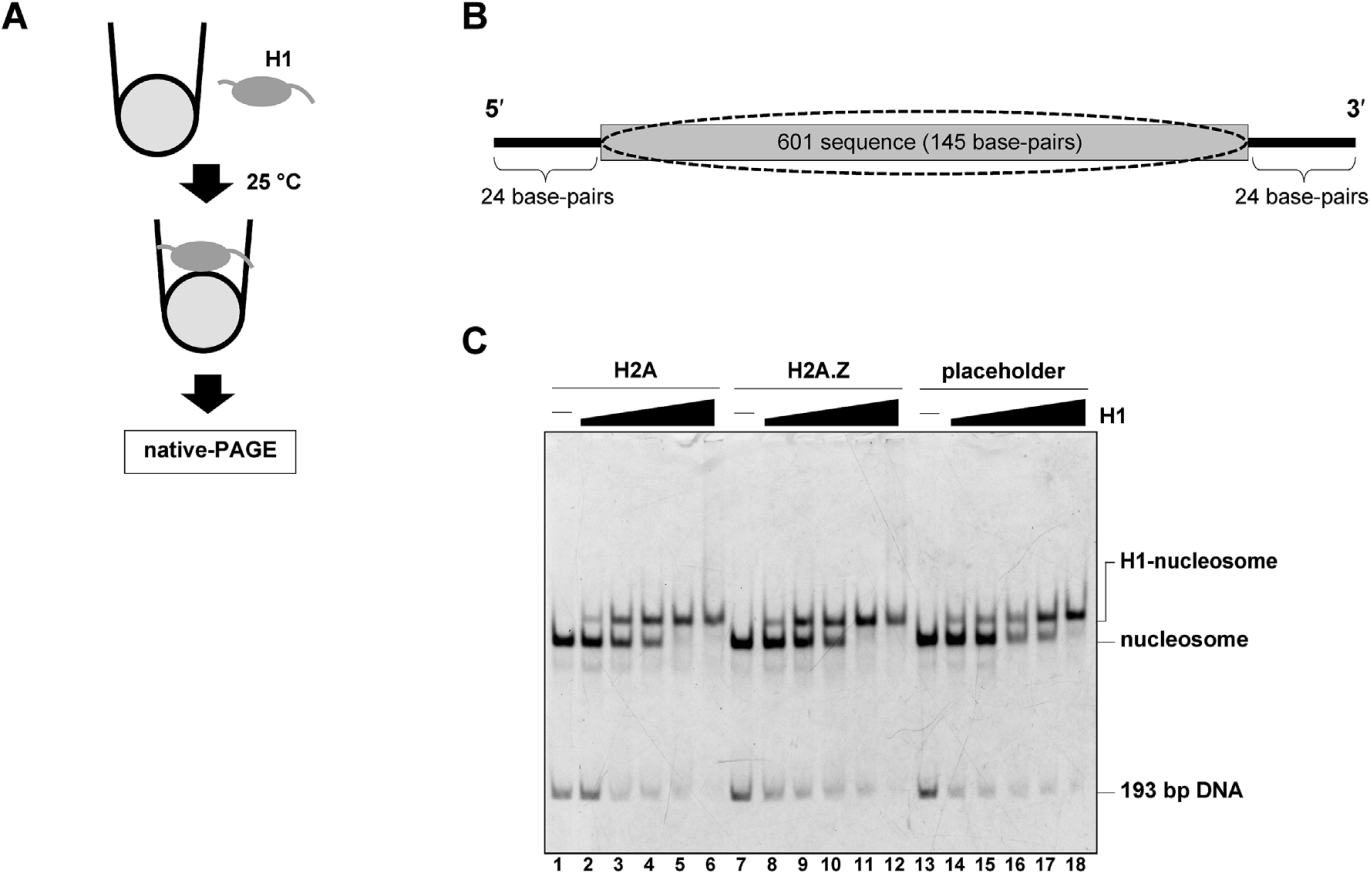
Linker histone H1 binding to the placeholder nucleosome. **A.** Schematic representation of the H1 binding assay. In this assay, increasing amounts of H1 are mixed with the nucleosomes in the presence of Nap1. After an incubation, the complex formation is analyzed by native-PAGE. **B.** The predicted nucleosome positioning on the 193 base-pair DNA is presented by an ellipse. A gray box indicates the 145 base-pair Widom 601 sequence region. **C.** A representative gel image of the H1 binding assay. Increasing amounts of H1 (0, 0.23, 0.32, 0.41, 0.50, and 0.60 μM) were mixed with the canonical (lanes 1–6), H2A.Z (lanes 7–12), and placeholder (lanes 13–18) nucleosomes (0.1 μM) containing the 193 base-pair Widom 601 DNA in the presence of Nap1 (0.3 μM). After an incubation at 25 °C for 30 min, the reaction mixtures were analyzed by native-PAGE with ethidium bromide staining.

The linker histone H1 may be involved in the recruitment of DNMT3B, which is a *de novo* eukaryotic DNA methyltransferase [28]. This suggested that the absence of the linker histone H1 in the placeholder nucleosome may restrict the DNMT3B recruitment to its target sites. To test this possibility, we performed the linker histone H1 binding assay (Fig. 4A and B). To clarify the difference, we performed the H1 binding assay in the presence of 70 mM NaCl, which weakens the H1-nucleosome interaction [20]. As shown in Fig. 4C, the linker histone H1 efficiently bound to the placeholder nucleosome, as well as the H2A.Z and canonical nucleosomes.

### 3.5 Perspective

In the present study, we performed biochemical analyses with the placeholder nucleosome containing H2A.Z and H3K4_C_me1, and found that the placeholder nucleosome conserves the characteristics of the H2A.Z nucleosome. This suggests that the placeholder nucleosome may function in the maintenance of genomic hypomethylation regions without the synergistic effects of H2A.Z and H3K4 methylation in the nucleosome structure. To maintain DNA hypomethylation, the placeholder nucleosome may inhibit DNA methyltransferase recruitment and/or activation. This may be accomplished by the H3K4 methylation, which inhibits the binding of *de novo* DNA methyltransferases, DNMT3A and DNMT3B, and their regulatory factor DNMT3L, to the H3 N-terminal tail [[29], [30], [31], [32]]. What is the role of H2A.Z in the placeholder nucleosome? The recruitment of the H3K4 methyltransferase, the MLL complex, may be promoted by the H2A.Z nucleosome in cells [33]. Therefore, the H2A.Z-mediated histone-methyltransferase recruitment may restrict the accumulation of DNA methyltransferases at the placeholder nucleosomes in cells.
